# Financial Barriers, Mental Health, and Medication Nonadherence Among Individuals With Chronic Pain

**DOI:** 10.1002/puh2.70345

**Published:** 2026-08-18

**Authors:** Zoe Sirotiak

**Affiliations:** ^1^ Department of Kinesiology and Health Iowa State University Ames Iowa USA

## Abstract

**Introduction:**

Chronic pain has been associated with comorbid psychological symptoms and prescription medication nonadherence, including nonadherence due to financial limitations. Despite noted independent associations with chronic pain, the association of medication nonadherence and psychological symptoms in the context of chronic pain is unclear. Therefore, this study aimed to explore the associations between depression and anxiety medication nonadherence due to cost among adults living with chronic pain.

**Method:**

Data from the 2023 National Health Interview Survey were utilized in these analyses. Adults (*N* = 7736) with self‐reported chronic pain responded to items assessing cost‐related medication nonadherence (skipping medication, taking less medication, delaying filling prescriptions, not getting prescription) and psychological symptoms (depression and anxiety).

**Results:**

Overall, 87.2% of individuals with chronic pain reported taking prescription medication during the past year. Skipping medication doses to save money, taking less medication to save money, delaying filling a prescription to save money, and not getting a prescription due to cost were each associated with depression and anxiety (all *p*s < 0.001) among individuals with chronic pain.

**Conclusions:**

Psychological symptoms and medication nonadherence due to cost are intricately related in the context of chronic pain. Screening individuals with chronic pain for medication adherence issues and psychological symptoms, as well as educating patients regarding applicable policies or programs for affordable medication access may be particularly important steps for increasing healthcare equity.

## 1 Introduction

Chronic pain, or pain that lasts for at least 3 months, has been associated with significant functional ability and quality of life limitations [1, 2]. Chronic pain can involve pain concentrated in one area or more generalized, widespread symptoms [3]. Chronic pain has been identified as a significant public health concern, with approximately 20.9% of adults in the United States estimated to have chronic pain [4]. High‐impact chronic pain is another significant concern, characterized by chronic pain that limits daily function and activities [4, 5]. Although chronic pain is recognized as a symptom of chronic diseases, such as diabetes and cancer, it has also been suggested as disease of its own [6].

Chronic pain has also been associated with psychological symptoms, including anxiety and depression. It has been estimated that 30%–60% of individuals with chronic pain may experience depression, and chronic pain and psychological symptoms are strongly associated with each other [7, 8]. It has been estimated that approximately 12 million Americans are currently living with comorbid chronic pain and clinically significant anxiety and depressive symptoms [9]. The comorbid status of these conditions can be particularly impactful, with nearly 70% of individuals with comorbid psychological symptoms and chronic pain reporting occupational limitations [9]. Chronic pain has also been identified as an independent risk factor for suicidality, with individuals with chronic pain having twice the risk of death by suicide compared to individuals without chronic pain [10, 11]. Syndemic theory has been utilized to describe how concurrent health conditions interact within a population, influenced by upstream social determinants of health [12]. Recent literature has considered a syndemic approach to chronic pain and mental health, finding greater syndemic burden is associated with worse outcomes, including quality of life scores [13]. Substantial cost‐related medication nonadherence has been noted among individuals with depression [14], indicating that individuals with both chronic pain and psychological symptoms may be particularly likely to experience cost‐related medication nonadherence and emphasizing the importance of considering these variables in tandem.

Given the significant impacts of chronic pain, identifying effective treatment and management strategies has been identified as an important public health objective [4]. Management strategies for chronic pain include prescription and over‐the‐counter medications, physical activity, physical therapy, psychological therapy, and alternative medicine techniques [15, 16, 17]. Frequently utilized over‐the‐counter medications for chronic pain include acetaminophen and nonsteroidal anti‐inflammatory drugs, whereas common prescription medications include antidepressant medications and antiepileptic medications [18]. Further complicating the management of chronic pain, it has been suggested that a significant proportion of individuals with chronic pain present with additional chronic diagnoses [19]. Despite the use of prescription medications to treat both chronic pain and comorbid conditions, medication adherence has been noted as a significant challenge in the context of chronic pain [20, 21]. Suggested reasons for medication nonadherence in the context of chronic pain include side effects, mistrust of healthcare providers, perceived non‐efficacy, and financial constraints [20, 22]. Financial stressors and chronic pain are intricately linked [23], indicating the importance of considering financial stressors in the context of chronic pain. Although mental health symptoms and medication nonadherence have been individually associated with chronic pain, it is unclear how these constructs may relate in the context of chronic pain. Despite the notable associations between chronic pain, psychological symptoms, and financial stressors, there is limited understanding of how financial barriers to medications may be associated with both medication nonadherence as well as mental health symptoms among individuals with chronic pain. Therefore, the purpose of this study is to explore the association between financial barriers to prescription medications and mental health symptoms among adults living with chronic pain.

## 2 Methods

Data from the 2023 National Health Interview Survey (NHIS) were utilized [24]. Adults (*N* = 29,404) residing in the United States took part in a face‐to‐face interview assessing sociodemographic and health variables. Of these participants, 7736 reported chronic pain and were included in these analyses. The National Center for Health Statistics Ethics Review Committee approved the 2023 NHIS and consent was provided by each participant prior to participation. Institutional Review Board approval of the author's home institution was not required as de‐identified NHIS data are publicly available online [24]. Participants in the 2023 NHIS reported sociodemographic and health characteristics, as well as health experiences variables, including prescription medication utilization as well as mental health and chronic pain status. Detailed sociodemographic characteristics of the sample are shown in Table 1.

**TABLE 1:** Sociodemographic characteristics of the sample.

Characteristic	Total *N* = 7736
Age, M ± SD	54.9 (17.4)
**Gender identity, *N* (%)**	
Male	3366 (46.7)
Female	4370 (53.3)
**Race**	
White	6224 (82.0)
Not White	1226 (18.0)
**Ethnicity**	
Not Hispanic or Latino	6930 (87.7)
Hispanic or Latino	806 (12.3)
**Education**	
High school or less	2952 (41.4)
More than high school	4754 (58.6)
**Income‐to‐poverty ratio**	
0.00–2.99	4212 (52.3)
≥3.00	3524 (47.7)
**Prescription medication**	
No	840 (12.8)
Yes	6889 (87.2)

*Note:* Unweighted *N*s reported. Percentages are weighted.

### 2.1 Measures

#### 2.1.1 Sociodemographic Characteristics

Participants reported age, sex, race, ethnicity, education level, and income‐to‐poverty line ratio. Age was top coded at 85 by the National Center for Health Statistics in the 2023 NHIS public use file to preserve confidentiality [24], so the age associated with participants ranged from 18 to 85.

#### 2.1.2 Chronic Pain Status

Participants responded to the item “In the past three months, how often did you have pain? Would you say never, some days, most days, or every day?” Participants endorsing either “most days” or “every day” were considered to have chronic pain, a definition utilized in previous literature [25, 26].

#### 2.1.3 Anxiety

The Generalized Anxiety Disorder‐2 (GAD‐2) [27] assessed anxiety status. Participants reported how often during the past 2 weeks they have been bothered by (1) feeling nervous, anxious, or on edge, and (2) not being able to stop or control worrying. Participants responded on a 4‐point Likert scale (0 = not at all, 3 = nearly every day). Responses were dichotomized into negative (total score of 0–2) or positive (total score of 3–6) scores, with a positive result indicating probable anxiety disorder [27]. Internal consistency of the scale was adequate (unweighted *α *= 0.93).

#### 2.1.4 Depression

The Patient Health Questionnaire‐2 (PHQ‐2) [28] was utilized to quantify depressive symptoms. Participants reported how often during the past 2 weeks they had been bothered by (1) little interest or pleasure in doing things, and (2) feeling down, depressed, or hopeless. Participants responded on a 4‐point Likert scale (0 = not at all, 3 = nearly every day). Participants were assigned either a negative (total score of 0–2) or positive (total score of 3–6) score, with a positive result indicating probable depressive disorder [28]. Internal consistency of the scale was adequate (unweighted *α *= 0.93).

#### 2.1.5 Medication Nonadherence

Participants were asked if at any time in the past 12 months they took prescription medication. Participants endorsing prescription medication use in the past 12 months were then asked several questions regarding utilization of these medications. Items included whether over the past 12 months an individual had: (1) skipped medication doses to save money; (2) took less medication to save money; and (3) delayed filling a prescription to save money. All participants were asked whether they needed prescription medication but did not get it because of cost. Participants responded “yes” or “no” to these items.

#### 2.1.6 Body Mass Index

Body mass index (BMI) was used categorically, as noted by the “BMICAT_A” variable in the 2023 NHIS codebook. This variable utilized the equation BMI = [Weight (kg)/Height (m^2^)]. Underweight (<18.5 kg/m^2^) and healthy weight individuals (18.5 to <25 kg/m^2^) were condensed due to low numbers within the underweight group. Other categories included overweight (≥25 to <30 kg/m^2^) and obese (≥30 kg/m^2^).

#### 2.1.7 Perceived Health

Participants responded to an item asking: “Would you say your health in general is excellent, very good, good, fair, or poor?” Perceived health was considered a factor variable and treated as such in the logistic regression analyses.

### 2.2 Statistical Analyses

R version 4.4.0 was utilized to conduct the analyses. Sampling weights and variance estimation variables were employed, in line with recommendations from the National Center for Health Statistics [24]. Analyses presented utilize the weights and variance estimation variables offered by the National Center for Health Statistics in the public use NHIS files [24]. Data coded as “refused,” “not ascertained,” or “don't know” were considered missing. Quasi‐binomial logistic regression utilizing the survey package adjustments assessed the association of a positive psychological screening (anxiety, depression) and medication utilization (skipped medication, took less medication, delayed filling prescription, did not get prescription) items. Individuals with a missing value in analyses were excluded from that analysis. For the skipped medication, took less medication, and delayed filling prescription models, participants were excluded if they reported no medication use in the past 12 months. Analyses accounted for sociodemographic characteristics (age, sex, race, ethnicity, education, income‐to‐poverty level), as well as BMI and perceived health status. The analytic sample used in the logistic regression models was as follows: anxiety (skipped medication *N* = 6463; less medication *N* = 6463; delayed medication *N* = 6463; did not get medication *N* = 7230) and depression (skipped medication *N* = 6465; less medication *N* = 6465; delayed medication *N* = 6465; did not get medication *N* = 7229). Demographics were coded as shown in Table 1.

## 3 Results

A total of 7736 participants endorsed chronic pain, with 6889 (weighted = 87.2%) of individuals with chronic pain also reporting prescription medication use. Participants were an average age of 54.9 (SD = 17.4) years, and most endorsed female (53.3%) identity. Participants were mostly White (82.0%) and non‐Hispanic (87.7%). Most participants reported more than high school education (58.6%). Approximately half of participants reported an income‐to‐poverty ratio between 0.00 and 2.99 (52.3%). Most (87.2%) reported taking a prescription medication over the past 12 months. Table 1 provides detailed sociodemographic characteristics.

Skipping medication, taking less medication, delaying filling a prescription, and not getting medication were each significantly associated with a positive anxiety screen. Individuals with a positive anxiety screen had 1.93 times the odds of skipping medication compared to individuals without a positive anxiety screen after accounting for sociodemographic and health‐related covariates (aOR = 1.93, 95% CI = 1.48–2.51, *p* < 0.001). In a comparison of proportions, 5.7% of individuals without anxiety endorsing skipping medications compared to 15.1% of individuals with anxiety. Individuals with a positive anxiety screen had 1.88 times the odds of reporting taking less medication than prescribed compared to individuals without a positive anxiety screen after accounting for sociodemographic and health‐related covariates (aOR = 1.88, 95% CI = 1.48–2.39, *p* < 0.001). In a comparison of proportions, 6.7% of individuals without anxiety endorsing skipping medications compared to 16.5% of individuals with anxiety. Individuals with a positive anxiety screen had 1.79 times the odds of reporting delaying taking medication compared to individuals without a positive anxiety screen after accounting for sociodemographic and health‐related covariates (aOR = 1.79, 95% CI = 1.41–2.28, *p* < 0.001). In a comparison of proportions, 8.5% of individuals without anxiety endorsing skipping medications compared to 20.5% of individuals with anxiety. Individuals with a positive anxiety screen had 2.08 times the odds of reporting not taking medication compared to individuals without a positive anxiety screen after accounting for sociodemographic and health‐related covariates (aOR = 2.08, 95% CI = 1.69–2.57, *p* < 0.001). In a comparison of proportions, 9.2% of individuals without anxiety endorsing skipping medications compared to 23.5% of individuals with anxiety. Table 2 indicates anxiety model results.

**TABLE 2:** Association between anxiety and medication adherence variables.

Predictor	*B* (SE)	*p* value	Odds ratio	95% CI
**Skipped medication**				
Positive anxiety screen	0.66 (0.13)	<0.001	1.93	1.48–2.51
Age, years	−0.02 (0.00)	<0.001	0.98	0.97–0.98
Sex: female	0.06 (0.12)	0.623	1.06	0.83–1.36
Race: not White	0.02 (0.15)	0.918	1.02	0.75–1.37
Ethnicity: Hispanic	−0.12 (0.22)	0.573	0.88	0.58–1.35
Education: ore than high school	0.12 (0.14)	0.390	1.13	0.86–1.49
Income‐to‐poverty ratio: 3.00 or higher	−0.69 (0.14)	<0.001	0.50	0.38–0.66
BMI: overweight	−0.05 (0.18)	0.796	0.95	0.67–1.36
BMI: obese	0.15 (0.17)	0.382	1.16	0.83–1.60
General health: fair/poor	0.62 (0.13)	<0.001	1.86	1.43–2.42
**Took less medication**				
Positive anxiety screen	0.63 (0.12)	<0.001	1.88	1.48–2.39
Age, years	−0.02 (0.00)	<0.001	0.98	0.97–0.99
Sex: female	0.12 (0.12)	0.298	1.13	0.90–1.43
Race: not White	0.01 (0.15)	0.942	1.01	0.76–1.35
Ethnicity: Hispanic	−0.21 (0.20)	0.300	0.81	0.55–1.20
Education: more than high school	0.16 (0.12)	0.204	1.17	0.92–1.49
Income‐to‐poverty ratio: 3.00 or higher	−0.71 (0.13)	<0.001	0.49	0.38–0.64
BMI: overweight	−0.02 (0.17)	0.911	0.98	0.71–1.36
BMI: obese	0.08 (0.15)	0.594	1.08	0.80–1.46
General health: fair/poor	0.57 (0.13)	<0.001	1.77	1.38–2.27
**Delayed filling a prescription**				
Positive anxiety screen	0.58 (0.12)	<0.001	1.79	1.41–2.28
Age, years	−0.03 (0.00)	<0.001	0.97	0.97–0.98
Sex: female	0.13 (0.11)	0.245	1.14	0.92–1.41
Race: not White	0.16 (0.13)	0.212	1.18	0.91–1.52
Ethnicity: Hispanic	−0.22 (0.20)	0.273	0.80	0.54–1.19
Education: more than high school	0.16 (0.12)	0.185	1.17	0.93–1.48
Income‐to‐poverty ratio: 3.00 or higher	−0.56 (0.12)	<0.001	0.57	0.45–0.73
BMI: overweight	0.07 (0.15)	0.661	1.07	0.79–1.45
BMI: obese	0.21 (0.15)	0.149	1.24	0.93–1.65
General health: fair/poor	0.58 (0.12)	<0.001	1.79	1.40–2.28
**Did not get needed medication**				
Positive anxiety screen	0.73 (0.11)	<0.001	2.08	1.69–2.57
Age, years	−0.02 (0.00)	<0.001	0.98	0.98–0.99
Sex: female	0.16 (0.09)	0.079	1.18	0.98–1.42
Race: not White	0.16 (0.11)	0.158	1.18	0.94–1.47
Ethnicity: Hispanic	−0.11 (0.17)	0.516	0.90	0.65–1.25
Education: more than high school	0.08 (0.10)	0.406	1.09	0.89–1.32
Income‐to‐poverty ratio: 3.00 or higher	−0.59 (0.11)	<0.001	0.55	0.44–0.69
BMI: overweight	0.32 (0.13)	0.017	1.37	1.06–1.78
BMI: obese	0.34 (0.13)	0.008	1.41	1.10–1.81
General health: fair/poor	0.68 (0.11)	<0.001	1.97	1.60–2.43

*Note:* Positive anxiety screen indicates a positive GAD‐2 screen. Models adjusted for age, sex, race, ethnicity, education, income‐to‐poverty ratio, body mass index, and perceived health. Skipped medication = skipped medication doses to save money in past 12 months. Less medication = took less medication to save money in past 12 months. Delayed medication = delayed filling prescription to save money in past 12 months. No medication = needed prescription medication but did not get it due to cost in past 12 month. The skipped medication, less medication, and delayed prescription models were restricted to prescription medication users.

Abbreviation: BMI, body mass index.

Skipping medication, taking less medication, delaying filling a prescription, and not getting medication were each significantly associated with a positive depression screen. Individuals with a positive depression screen had 2.02 times the odds of skipping medication compared to individuals without a positive depression screen after accounting for sociodemographic and health‐related covariates (aOR = 2.02, 95% CI = 1.55–2.64, *p* < 0.001). In a comparison of proportions, 6.0% of individuals without depression endorsing skipping medications compared to 15.4% of individuals with depression. Individuals with a positive depression screen had 1.74 times the odds of reporting taking less medication than prescribed compared to individuals without a depression anxiety screen after accounting for sociodemographic and health‐related covariates (aOR = 1.74, 95% CI = 1.36–2.23, *p* < 0.001). In a comparison of proportions, 7.2% of individuals without depression endorsing skipping medications compared to 15.8% of individuals with depression. Individuals with a positive depression screen had 1.74 times the odds of reporting delaying taking medication compared to individuals without a positive depression screen after accounting for sociodemographic and health‐related covariates (aOR = 1.74, 95% CI = 1.37–2.22, *p* < 0.001). In a comparison of proportions, 9.1% of individuals without depression endorsing skipping medications compared to 20.0% of individuals with depression. Individuals with a positive depression screen had 1.98 times the odds of reporting not taking medication compared to individuals without a positive depression screen after accounting for sociodemographic and health‐related covariates (aOR = 1.98, 95% CI = 1.59–2.48, *p* < 0.001). In a comparison of proportions, 9.7% of individuals without depression endorsing skipping medications compared to 23.8% of individuals with depression. Table 3 indicates depression model results.

**TABLE 3:** Association between depression and medication adherence variables.

Predictor	*B* (SE)	*p* value	Odds ratio	95% CI
**Skipped medication**				
Positive depression screen	0.70 (0.14)	<0.001	2.02	1.55–2.64
Age, years	−0.03 (0.00)	<0.001	0.97	0.97–0.98
Sex: female	0.11 (0.12)	0.375	1.12	0.88–1.42
Race: not White	−0.05 (0.15)	0.750	0.95	0.71–1.29
Ethnicity: Hispanic	−0.16 (0.22)	0.473	0.86	0.56–1.31
Education: more than high school	0.12 (0.14)	0.394	1.13	0.86–1.48
Income‐to‐poverty ratio: 3.00 or higher	−0.67 (0.14)	<0.001	0.51	0.39–0.67
BMI: overweight	−0.07 (0.18)	0.684	0.93	0.65–1.33
BMI: obese	0.14 (0.17)	0.410	1.15	0.83–1.59
General health: fair/poor	0.60 (0.14)	<0.001	1.83	1.40–2.38
**Took less medication**				
Positive depression screen	0.56 (0.13)	<0.001	1.74	1.36–2.23
Age, years	−0.02 (0.00)	<0.001	0.98	0.97–0.98
Sex: female	0.17 (0.12)	0.153	1.18	0.94–1.49
Race: not White	−0.05 (0.15)	0.738	0.95	0.71–1.27
Ethnicity: Hispanic	−0.23 (0.20)	0.243	0.79	0.54–1.17
Education: more than high school	0.15 (0.12)	0.217	1.16	0.91–1.48
Income‐to‐poverty ratio: 3.00 or higher	−0.71 (0.13)	<0.001	0.49	0.38–0.64
BMI: overweight	−0.04 (0.17)	0.796	0.96	0.69–1.33
BMI: obese	0.07 (0.15)	0.647	1.07	0.79–1.45
General health: fair/poor	0.58 (0.13)	<0.001	1.78	1.39–2.28
**Delayed filling a prescription**				
Positive depression screen	0.55 (0.12)	<0.001	1.74	1.37–2.22
Age, years	−0.03 (0.00)	<0.001	0.97	0.97–0.98
Sex: female	0.17 (0.11)	0.126	1.18	0.95–1.47
Race: not White	0.13 (0.13)	0.315	1.14	0.88–1.48
Ethnicity: Hispanic	−0.25 (0.20)	0.207	0.78	0.53–1.15
Education: more than high school	0.15 (0.12)	0.209	1.16	0.92–1.46
Income‐to‐poverty ratio: 3.00 or higher	−0.55 (0.12)	<0.001	0.58	0.45–0.73
BMI: overweight	0.04 (0.15)	0.794	1.04	0.77–1.41
BMI: obese	0.19 (0.15)	0.196	1.21	0.91–1.61
General health: fair/poor	0.59 (0.12)	<0.001	1.80	1.41–2.29
**Did not get needed medication**				
Positive depression screen	0.69 (0.11)	<0.001	1.98	1.59–2.48
Age, years	−0.02 (0.00)	<0.001	0.98	0.97–0.98
Sex: female	0.21 (0.09)	0.026	1.23	1.03–1.49
Race: not White	0.12 (0.12)	0.317	1.12	0.89–1.41
Ethnicity: Hispanic	−0.17 (0.17)	0.322	0.85	0.61–1.18
Education: more than high school	0.07 (0.10)	0.453	1.08	0.89–1.31
Income‐to‐poverty ratio: 3.00 or higher	−0.58 (0.11)	<0.001	0.56	0.45–0.69
BMI: overweight	0.30 (0.13)	0.025	1.35	1.04–1.75
BMI: obese	0.33 (0.13)	0.011	1.39	1.08–1.79
General health: fair/poor	0.70 (0.11)	<0.001	2.02	1.65–2.49

*Note:* Positive depression screen indicates a positive PHQ‐2 screen. Models adjusted for age, sex, race, ethnicity, education, income‐to‐poverty ratio, body mass index, and perceived health. Skipped medication = skipped medication doses to save money in past 12 months. Less medication = took less medication to save money in past 12 months. Delayed medication = delayed filling prescription to save money in past 12 months. No medication = needed prescription medication but did not get it due to cost in past 12 month. The skipped medication, less medication, and delayed prescription models were restricted to prescription medication users.

Abbreviation: BMI, body mass index.

## 4 Discussion

Chronic pain is associated with mental health symptoms and medication adherence barriers [7, 8, 20, 21], but it is unclear how mental health symptoms and medication adherence may relate in the context of chronic pain. Therefore, the purpose of this study was to assess the associations between depression and anxiety and medication nonadherence due to financial cost in the context of chronic pain.

Mental health symptoms and poor medication adherence appear to be consistently associated in individuals with chronic pain, aligning with past literature indicating associations between these variables [13, 14, 29]. Financial barriers appear to contribute substantially to this relationship, with skipping medication due to cost, taking less medication due to cost, delaying filling prescription due to cost, and not getting medications because of cost each significantly associated with depression and anxiety in the context of chronic pain, after accounting for sociodemographic and health‐related variables. This finding highlights the importance of considering prescription medication adherence among individuals with chronic pain, particularly among individuals with comorbid mental health symptoms and diagnoses. With substantial proportions of individuals with chronic pain also noting comorbid mental health symptoms [7, 8, 9], considering the associations between prescription medication adherence and mental health in the context of chronic pain appears to be particularly important.

Financial limitations have been frequently noted in the chronic pain population [23], indicating that considering financial barriers to prescription medication adherence may be particularly relevant in the context of chronic pain. Socioeconomic status has been previously associated with chronic pain, with individuals with low or medium socioeconomic status experiencing a moderately increased risk of chronic pain compared to individuals of high socioeconomic status [30]. Social inequalities have been noted to impact chronic pain among both men and women, emphasizing the importance of considering the social context of individuals experiencing chronic pain [31]. The significant associations between prescription medication nonadherence and each mental health symptom noted indicates the increased impact that individuals with chronic pain may face. Although chronic pain has been individually associated with medication nonadherence and psychological symptoms [7, 8, 9, 20, 21], it appears that these variables may also relate to each other in the context of chronic pain indicating potentially compounded inequalities among individuals with chronic pain.

The notable associations between psychological symptoms and cost‐related medication nonadherence among individuals with chronic pain in this analysis lend themselves to interpretation through a syndemic perspective. Chronic pain and psychological symptoms have been independently associated with financial strain [32, 33], and it appears that cost‐related medication barriers may be particularly important to consider among individuals with these conditions presenting comorbidly. Although past literature has demonstrated the associations between psychological symptoms and cost‐related medication nonadherence [29], these analyses expand our understanding of this association by focusing specifically on chronic pain, one of the most prevalent chronic health challenges.

Given these findings, healthcare clinicians working with individuals with chronic pain may benefit from assessing medication adherence and psychological symptoms among patients with chronic pain. The substantial proportion of individuals with chronic pain noting prescription medication nonadherence because of financial limitations indicates that providing prescriptions and instructing patients how and when to take medications may not be enough to ensure adherence; in addition, assessing financial barriers to medication adherence may be particularly beneficial. The associations identified also indicate the importance of screening for both chronic pain and mental health symptoms among individuals with the other symptom. Chronic pain is often underdiagnosed or misdiagnosed, resulting in a lack of effective treatment [34, 35]. Individuals with chronic pain often go prolonged periods of time without a diagnosis, and substantial psychological distress is common [34, 36, 37]. Assessing for chronic pain among individuals with psychological symptoms, as well as probing for and treating psychological symptoms among individuals with chronic pain disorders may be particularly important in improving quality of life and health outcomes. Clinicians should be aware of prescription medication cost, as well as applicable policies and programs aimed at reducing the cost of prescription medications. The results of this study indicate that educating patients regarding these policies and programs may provide particular benefit.

This study had several limitations. All data were cross‐sectional, and temporal associations cannot be determined. In addition, all variables were self‐reported, and severity and duration of chronic pain were not considered in these analyses, though it is possible these variables may influence the experience of participants. It is unclear what prescription medications participants may have been taking, limiting interpretation regarding whether medication nonadherence was specifically related to chronic pain treatment. Additionally, the duration of medication use was not assessed in this study, though duration of use may influence the financial burden as well as mental health of the individual affected. Cost‐related medication nonadherence items were assessed over the past 12 months, whereas anxiety and depression were measured over the past 2 weeks. Therefore, the constructs are measured over different periods of time and combined with the cross‐sectional nature of the data, temporal order of the constructs cannot be determined. Participants were mostly White and non‐Hispanic, and it is unclear how the experience of medication adherence and psychological symptoms may differ in other racial and ethnic groups. Finally, all participants were adults in the United States, and it is unclear how the experience of other populations may differ.

In conclusion, prescription medication adherence and psychological symptoms appear to be intricately related in the context of chronic pain, emphasizing the importance of screening for medication adherence and psychological symptoms in the context of chronic pain. Financial barriers to medication adherence appear to be particularly prevalent in the context of chronic pain, emphasizing the importance of screening for financial limitations to prescription medication use among individuals with chronic pain. Healthcare providers may increase awareness of medication access limitations, as well as applicable policies and programs aimed at reducing the cost of prescription medications for their patients. Future studies may use longitudinal methods to identify temporal associations, aiding in intervention development. In addition, qualitative analyses considering the prescription medication access experiences of individuals with chronic pain may further illuminate associations between medication access and mental health symptoms among individuals with chronic pain.

## Author Contributions

**Zoe Sirotiak**: conceptualization, formal analysis, writing – original draft preparation, writing – reviewing and editing.

## Funding

The author has nothing to report.

## Ethics Statement

No human participants or animals were utilized in experiments performed by the author. The current study is a secondary data analysis of an open access, de‐identified dataset from the National Interview Health Survey (NHIS). The Ethics Review Board of the National Center for Health Statistics approved and oversaw the 2023 NHIS.

## Conflicts of Interest

The author declares no conflicts of interest.

## Supporting information




**Supporting file 1**: puh270345‐sup‐0001‐SuppMat.pdf

## Data Availability

2023 NHIS public use data is publicly available at https://www.cdc.gov/nchs/nhis/documentation/2023‐nhis.html?CDC_AAref_Val=https://www.cdc.gov/nchs/nhis/2023nhis.htm. Full code is available in the Supporting Information.
