# Supplementary material for: Financial Barriers, Mental Health, and Medication Nonadherence Among Individuals With Chronic Pain
Source: Public Health Chall. 2026 Aug 18;5(3):e70345. doi: 10.1002/puh2.70345 (PMC13484898; doi:10.1002/puh2.70345)
Supplement: Supplementary file 1 [file PUH2-5-e70345-s001.pdf]

Financial barriers, mental health, and medication nonadherence among individuals with chronic pain

2026-07-31

```
### Load packages
library(dplyr)
library(survey)
library(broom)
library(gt)

### Start with the original NHIS dataset
data <- NHIS_2023

### Derive all analysis variables from original NHIS variables
data <- data %>%
  mutate(

    #--------------------------------------------------------------------------
    # Age
    # AGEP_A:
    # 18-85 = valid age
    # 85 is top coded as 85+
    # 97-99 = missing responses
    #--------------------------------------------------------------------------

    age = case_when(
      AGEP_A >= 18 & AGEP_A <= 85 ~ as.numeric(AGEP_A),
      TRUE ~ NA_real_
    ),


    #--------------------------------------------------------------------------
    # Chronic pain
    #
    # PAIFRQ3M_A:
    # 1 = Never
    # 2 = Some days
    # 3 = Most days
    # 4 = Every day
    #
    # Chronic pain = most days or every day
    #--------------------------------------------------------------------------

    chrpain = case_when(
      PAIFRQ3M_A %in% c(3, 4) ~ 1,
      PAIFRQ3M_A %in% c(1, 2) ~ 0,
      TRUE ~ NA_real_
```

```r
  ),


  #-----------------------------------------------------------------------------
  # Depression screen
  #
  # PHQ2SCREEN_A:
  # 1 = Positive
  # 2 = Negative
  # 8 = Not ascertained
  #
  # Negative is the reference group.
  #-----------------------------------------------------------------------------

  phqscreen = case_when(
    PHQ2SCREEN_A == 1 ~ "Positive",
    PHQ2SCREEN_A == 2 ~ "Negative",
    TRUE ~ NA_character_
  ),

  phqscreen = factor(
    phqscreen,
    levels = c(
      "Negative",
      "Positive"
    )
  ),


  #-----------------------------------------------------------------------------
  # Anxiety screen
  #
  # GAD2SCREEN_A:
  # 1 = Positive
  # 2 = Negative
  # 8 = Not ascertained
  #
  # Negative is the reference group.
  #-----------------------------------------------------------------------------

  gadscreen = case_when(
    GAD2SCREEN_A == 1 ~ "Positive",
    GAD2SCREEN_A == 2 ~ "Negative",
    TRUE ~ NA_character_
  ),

  gadscreen = factor(
    gadscreen,
    levels = c(
      "Negative",
      "Positive"
    )
  ),
```

```r
#-------------------------------------------------------------------------------
# Sex
#
# SEX_A:
# 1 = Male
# 2 = Female
#
# Male is the reference group.
#-------------------------------------------------------------------------------

sex = case_when(
  SEX_A == 1 ~ "Male",
  SEX_A == 2 ~ "Female",
  TRUE ~ NA_character_
),

sex = factor(
  sex,
  levels = c(
    "Male",
    "Female"
  )
),


#-------------------------------------------------------------------------------
# Race
#
# RACEALLP_A:
# 1 = White
# 2-6 = all other reported race groups
# 7-9 = missing responses
#
# White is the reference group.
#-------------------------------------------------------------------------------

race_dichot = case_when(
  RACEALLP_A == 1 ~ "White only",
  RACEALLP_A %in% 2:6 ~ "All other races",
  TRUE ~ NA_character_
),

race_dichot = factor(
  race_dichot,
  levels = c(
    "White only",
    "All other races"
  )
),


#-------------------------------------------------------------------------------
```

```r
# Ethnicity
#
# HISP_A:
# 1 = Hispanic
# 2 = Not Hispanic
#
# Non-Hispanic is the reference group.
#----------------------------------------------------------------------------

ethnicity = case_when(
  HISP_A == 1 ~ "Hispanic or Latino",
  HISP_A == 2 ~ "Not Hispanic or Latino",
  TRUE ~ NA_character_
),

ethnicity = factor(
  ethnicity,
  levels = c(
    "Not Hispanic or Latino",
    "Hispanic or Latino"
  )
),


#----------------------------------------------------------------------------
# Education
#
# EDUCP_A:
# 0-4 = high school or less
# 5-10 = more than high school
# 97-99 = missing responses
#
# High school or less is the reference group.
#----------------------------------------------------------------------------

education = case_when(
  EDUCP_A %in% 0:4 ~ "High school or less",
  EDUCP_A %in% 5:10 ~ "More than high school",
  TRUE ~ NA_character_
),

education = factor(
  education,
  levels = c(
    "High school or less",
    "More than high school"
  )
),


#----------------------------------------------------------------------------
# Income-to-poverty ratio
#
```

```r
# RATCAT_A:
# 1-9 = 0.00-2.99
# 10-14 = 3.00 or higher
#----------------------------------------------------------------------------

ratcat = case_when(
  RATCAT_A %in% 1:9 ~ "0.00-2.99",
  RATCAT_A %in% 10:14 ~ "3.00 or higher",
  TRUE ~ NA_character_
),

ratcat = factor(
  ratcat,
  levels = c(
    "0.00-2.99",
    "3.00 or higher"
  )
),


#----------------------------------------------------------------------------
# BMI
#
# BMICAT_A:
# 1 = Underweight
# 2 = Healthy weight
# 3 = Overweight
# 4 = Obese
#----------------------------------------------------------------------------

bmi = case_when(
  BMICAT_A %in% c(1, 2) ~ "Underweight/Healthy",
  BMICAT_A == 3 ~ "Overweight",
  BMICAT_A == 4 ~ "Obese",
  TRUE ~ NA_character_
),

bmi = factor(
  bmi,
  levels = c(
    "Underweight/Healthy",
    "Overweight",
    "Obese"
  )
),


#----------------------------------------------------------------------------
# Perceived health
#
# PHSTAT_A:
# 1 = Excellent
# 2 = Very good
```

```r
  # 3 = Good
  # 4 = Fair
  # 5 = Poor
  #------------------------------------------------------------------------------

  health_binary = case_when(
    PHSTAT_A %in% 1:3 ~ "Good/Very Good/Excellent",
    PHSTAT_A %in% 4:5 ~ "Fair/Poor",
    TRUE ~ NA_character_
  ),

  health_binary = factor(
    health_binary,
    levels = c(
      "Good/Very Good/Excellent",
      "Fair/Poor"
    )
  ),


  #------------------------------------------------------------------------------
  # Prescription medication use
  #
  # RX12M_A:
  # 1 = Yes
  # 2 = No
  #------------------------------------------------------------------------------

  prescription_yn = case_when(
    RX12M_A == 1 ~ 1,
    RX12M_A == 2 ~ 0,
    TRUE ~ NA_real_
  ),


  #------------------------------------------------------------------------------
  # Skipped medication to save money
  #
  # RXSK12M_A:
  # 1 = Yes
  # 2 = No
  #------------------------------------------------------------------------------

  skipmed = case_when(
    RXSK12M_A == 1 ~ 1,
    RXSK12M_A == 2 ~ 0,
    TRUE ~ NA_real_
  ),


  #------------------------------------------------------------------------------
  # Took less medication to save money
  #
```

```
    # RXLS12M_A:
    # 1 = Yes
    # 2 = No
    #---------------------------------------------------------------------------

    lessmed = case_when(
      RXLS12M_A == 1 ~ 1,
      RXLS12M_A == 2 ~ 0,
      TRUE ~ NA_real_
    ),


    #---------------------------------------------------------------------------
    # Delayed filling a prescription to save money
    #
    # RXDL12M_A:
    # 1 = Yes
    # 2 = No
    #---------------------------------------------------------------------------

    delaymed = case_when(
      RXDL12M_A == 1 ~ 1,
      RXDL12M_A == 2 ~ 0,
      TRUE ~ NA_real_
    ),


    #---------------------------------------------------------------------------
    # Needed medication but did not get it because of cost
    #
    # RXDG12M_A:
    # 1 = Yes
    # 2 = No
    #---------------------------------------------------------------------------

    nomed = case_when(
      RXDG12M_A == 1 ~ 1,
      RXDG12M_A == 2 ~ 0,
      TRUE ~ NA_real_
    )
  )


### Check all derived variables
table(data$chrpain, useNA = "ifany")
table(data$phqscreen, useNA = "ifany")
table(data$gadscreen, useNA = "ifany")
table(data$sex, useNA = "ifany")
table(data$race_dichot, useNA = "ifany")
table(data$ethnicity, useNA = "ifany")
table(data$education, useNA = "ifany")
table(data$ratcat, useNA = "ifany")
table(data$bmi, useNA = "ifany")
```

```r
table(data$health_binary, useNA = "ifany")

table(data$prescription_yn, useNA = "ifany")
table(data$skipmed, useNA = "ifany")
table(data$lessmed, useNA = "ifany")
table(data$delaymed, useNA = "ifany")
table(data$nomed, useNA = "ifany")

### Create the NHIS survey design
nhissvy <- svydesign(
  id = ~PPSU,
  strata = ~PSTRAT,
  weights = ~WTFA_A,
  data = data,
  nest = TRUE
)

# All adults with chronic pain
chronic_pain_design <- subset(
  nhissvy,
  chrpain == 1
)


# Adults with chronic pain who used prescription medication
medication_user_design <- subset(
  nhissvy,
  chrpain == 1 &
    prescription_yn == 1
)


### TABLE 1: ALL ADULTS WITH CHRONIC PAIN
# N = unweighted
# Percentages, mean, and SD = weighted
total_n <- nrow(
  chronic_pain_design$variables
)

# Age
age_mean <- as.numeric(
  coef(
    svymean(
      ~age,
      design = chronic_pain_design,
      na.rm = TRUE
    )
  )
)

age_sd <- sqrt(
  as.numeric(
    coef(
```

```r
      svyvar(
        ~age,
        design = chronic_pain_design,
        na.rm = TRUE
      )
    )
  )
)

age_row <- data.frame(
  Variable = "Age",
  Category = "Mean (SD), years",
  Unweighted_N = sum(
    !is.na(chronic_pain_design$variables$age)
  ),
  Weighted_Result = paste0(
    round(age_mean, 1),
    " (",
    round(age_sd, 1),
    ")"
  )
)

# Sex
sex_n <- table(
  chronic_pain_design$variables$sex
)

sex_pct <- prop.table(
  svytable(
    ~sex,
    design = chronic_pain_design
  )
) * 100

sex_rows <- data.frame(
  Variable = "Sex",
  Category = names(sex_n),
  Unweighted_N = as.numeric(sex_n),
  Weighted_Result = paste0(
    round(as.numeric(sex_pct), 1),
    "%"
  )
)

# Race
race_n <- table(
  chronic_pain_design$variables$race_dichot
)

race_pct <- prop.table(
  svytable(
    ~race_dichot,
```

```
    design = chronic_pain_design
  )
) * 100

race_rows <- data.frame(
  Variable = "Race",
  Category = names(race_n),
  Unweighted_N = as.numeric(race_n),
  Weighted_Result = paste0(
    round(as.numeric(race_pct), 1),
    "%"
  )
)

# Ethnicity
ethnicity_n <- table(
  chronic_pain_design$variables$ethnicity
)

ethnicity_pct <- prop.table(
  svytable(
    ~ethnicity,
    design = chronic_pain_design
  )
) * 100

ethnicity_rows <- data.frame(
  Variable = "Ethnicity",
  Category = names(ethnicity_n),
  Unweighted_N = as.numeric(ethnicity_n),
  Weighted_Result = paste0(
    round(as.numeric(ethnicity_pct), 1),
    "%"
  )
)

# Education
education_n <- table(
  chronic_pain_design$variables$education
)

education_pct <- prop.table(
  svytable(
    ~education,
    design = chronic_pain_design
  )
) * 100

education_rows <- data.frame(
  Variable = "Education",
  Category = names(education_n),
  Unweighted_N = as.numeric(education_n),
  Weighted_Result = paste0(
```

```
    round(as.numeric(education_pct), 1),
    "%"
  )
)

# Income-to-poverty ratio
ratcat_n <- table(
  chronic_pain_design$variables$ratcat
)

ratcat_pct <- prop.table(
  svytable(
    ~ratcat,
    design = chronic_pain_design
  )
) * 100

ratcat_rows <- data.frame(
  Variable = "Income-to-poverty ratio",
  Category = names(ratcat_n),
  Unweighted_N = as.numeric(ratcat_n),
  Weighted_Result = paste0(
    round(as.numeric(ratcat_pct), 1),
    "%"
  )
)

# Prescription medication use
chronic_pain_design <- update(
  chronic_pain_design,

  prescription_use = factor(
    prescription_yn,
    levels = c(0, 1),
    labels = c("No", "Yes")
  )
)

prescription_n <- table(
  chronic_pain_design$variables$prescription_use
)

prescription_pct <- prop.table(
  svytable(
    ~prescription_use,
    design = chronic_pain_design
  )
) * 100

prescription_rows <- data.frame(
  Variable = "Prescription medication",
  Category = names(prescription_n),
  Unweighted_N = as.numeric(prescription_n),
```

```r
  Weighted_Result = paste0(
    round(as.numeric(prescription_pct), 1),
    "%"
  )
)

# Combine Table 1
table1 <- bind_rows(
  age_row,
  sex_rows,
  race_rows,
  ethnicity_rows,
  education_rows,
  ratcat_rows,
  prescription_rows
)

table1

# Format and export Table 1
table1_gt <- table1 %>%
  gt(
    groupname_col = "Variable"
  ) %>%

  cols_label(
    Category = "",
    Unweighted_N = "Unweighted N",
    Weighted_Result = "Weighted % or M (SD)"
  ) %>%

  tab_header(
    title = "Table 1. Characteristics of Adults With Chronic Pain",
    subtitle = paste0(
      "Unweighted N = ",
      format(total_n, big.mark = ",")
    )
  ) %>%

  tab_source_note(
    source_note = paste(
      "Note. Ns are unweighted.",
      "Percentages, means, and standard deviations are survey weighted.",
      "Percentages are based on participants with nonmissing data",
      "for the corresponding characteristic."
    )
  )

table1_gt

gtsave(
  table1_gt,
  "Table1_Chronic_Pain_Characteristics.docx"
```

```r
)

### ANXIETY MODELS

anx_skip_model <- svyglm(
  skipmed ~ gadscreen + age + sex + race_dichot +
    ethnicity + education + ratcat + bmi + health_binary,
  design = medication_user_design,
  family = quasibinomial()
)

anx_less_model <- svyglm(
  lessmed ~ gadscreen + age + sex + race_dichot +
    ethnicity + education + ratcat + bmi + health_binary,
  design = medication_user_design,
  family = quasibinomial()
)

anx_delay_model <- svyglm(
  delaymed ~ gadscreen + age + sex + race_dichot +
    ethnicity + education + ratcat + bmi + health_binary,
  design = medication_user_design,
  family = quasibinomial()
)

# This outcome was asked of all adults
anx_nomed_model <- svyglm(
  nomed ~ gadscreen + age + sex + race_dichot +
    ethnicity + education + ratcat + bmi + health_binary,
  design = chronic_pain_design,
  family = quasibinomial()
)

### DEPRESSION MODELS

dep_skip_model <- svyglm(
  skipmed ~ phqscreen + age + sex + race_dichot +
    ethnicity + education + ratcat + bmi + health_binary,
  design = medication_user_design,
  family = quasibinomial()
)

dep_less_model <- svyglm(
  lessmed ~ phqscreen + age + sex + race_dichot +
    ethnicity + education + ratcat + bmi + health_binary,
  design = medication_user_design,
  family = quasibinomial()
)

dep_delay_model <- svyglm(
  delaymed ~ phqscreen + age + sex + race_dichot +
    ethnicity + education + ratcat + bmi + health_binary,
  design = medication_user_design,
```

```
  family = quasibinomial()
)

# This outcome was asked of all adults
dep_nomed_model <- svyglm(
  nomed ~ phqscreen + age + sex + race_dichot +
    ethnicity + education + ratcat + bmi + health_binary,
  design = chronic_pain_design,
  family = quasibinomial()
)

### Check actual model sample sizes

nrow(model.frame(anx_skip_model))
nrow(model.frame(anx_less_model))
nrow(model.frame(anx_delay_model))
nrow(model.frame(anx_nomed_model))

nrow(model.frame(dep_skip_model))
nrow(model.frame(dep_less_model))
nrow(model.frame(dep_delay_model))
nrow(model.frame(dep_nomed_model))

### PREPARE TABLE 2: ANXIETY

anx_skip_results <- tidy(
  anx_skip_model,
  conf.int = TRUE
)

anx_skip_results$Outcome <- "Skipped medication"


anx_less_results <- tidy(
  anx_less_model,
  conf.int = TRUE
)

anx_less_results$Outcome <- "Took less medication"


anx_delay_results <- tidy(
  anx_delay_model,
  conf.int = TRUE
)

anx_delay_results$Outcome <- "Delayed filling a prescription"


anx_nomed_results <- tidy(
  anx_nomed_model,
  conf.int = TRUE
)
```

```r
anx_nomed_results$Outcome <- "Did not get needed medication"


anxiety_table <- bind_rows(
  anx_skip_results,
  anx_less_results,
  anx_delay_results,
  anx_nomed_results
) %>%
  filter(term != "(Intercept)") %>%
  mutate(

    Predictor = case_when(
      term == "gadscreenPositive" ~ "Positive anxiety screen",
      term == "age" ~ "Age, years",

      grepl("^sex", term) ~
        sub("^sex", "Sex: ", term),

      grepl("^race_dichot", term) ~
        sub("^race_dichot", "Race: ", term),

      grepl("^ethnicity", term) ~
        sub("^ethnicity", "Ethnicity: ", term),

      grepl("^education", term) ~
        sub("^education", "Education: ", term),

      grepl("^ratcat", term) ~
        sub(
          "^ratcat",
          "Income-to-poverty ratio: ",
          term
        ),

      grepl("^bmi", term) ~
        sub("^bmi", "BMI: ", term),

      grepl("^health_binary", term) ~
        sub(
          "^health_binary",
          "General health: ",
          term
        ),

      TRUE ~ term
    ),

    `B (SE)` = paste0(
      sprintf("%.2f", estimate),
      " (",
      sprintf("%.2f", std.error),
      ")"
```

```
    ),

    `p-value` = ifelse(
      p.value < 0.001,
      "<0.001",
      sprintf("%.3f", p.value)
    ),

    `Odds ratio` = sprintf(
      "%.2f",
      exp(estimate)
    ),

    `95% CI` = paste0(
      sprintf("%.2f", exp(conf.low)),
      "-",
      sprintf("%.2f", exp(conf.high))
    )
  ) %>%
  select(
    Outcome,
    Predictor,
    `B (SE)`,
    `p-value`,
    `Odds ratio`,
    `95% CI`
  )

anxiety_table

### PREPARE TABLE 3: DEPRESSION

dep_skip_results <- tidy(
  dep_skip_model,
  conf.int = TRUE
)

dep_skip_results$Outcome <- "Skipped medication"


dep_less_results <- tidy(
  dep_less_model,
  conf.int = TRUE
)

dep_less_results$Outcome <- "Took less medication"


dep_delay_results <- tidy(
  dep_delay_model,
  conf.int = TRUE
)
```

```r
dep_delay_results$Outcome <- "Delayed filling a prescription"


dep_nomed_results <- tidy(
  dep_nomed_model,
  conf.int = TRUE
)

dep_nomed_results$Outcome <- "Did not get needed medication"


depression_table <- bind_rows(
  dep_skip_results,
  dep_less_results,
  dep_delay_results,
  dep_nomed_results
) %>%
  filter(term != "(Intercept)") %>%
  mutate(

    Predictor = case_when(
      term == "phqscreenPositive" ~ "Positive depression screen",
      term == "age" ~ "Age, years",

      grepl("^sex", term) ~
        sub("^sex", "Sex: ", term),

      grepl("^race_dichot", term) ~
        sub("^race_dichot", "Race: ", term),

      grepl("^ethnicity", term) ~
        sub("^ethnicity", "Ethnicity: ", term),

      grepl("^education", term) ~
        sub("^education", "Education: ", term),

      grepl("^ratcat", term) ~
        sub(
          "^ratcat",
          "Income-to-poverty ratio: ",
          term
        ),

      grepl("^bmi", term) ~
        sub("^bmi", "BMI: ", term),

      grepl("^health_binary", term) ~
        sub(
          "^health_binary",
          "General health: ",
          term
        ),
```

```r
      TRUE ~ term
    ),

    `B (SE)` = paste0(
      sprintf("%.2f", estimate),
      " (",
      sprintf("%.2f", std.error),
      ")"
    ),

    `p-value` = ifelse(
      p.value < 0.001,
      "<0.001",
      sprintf("%.3f", p.value)
    ),

    `Odds ratio` = sprintf(
      "%.2f",
      exp(estimate)
    ),

    `95% CI` = paste0(
      sprintf("%.2f", exp(conf.low)),
      "-",
      sprintf("%.2f", exp(conf.high))
    )
  ) %>%
  select(
    Outcome,
    Predictor,
    `B (SE)`,
    `p-value`,
    `Odds ratio`,
    `95% CI`
  )

depression_table

### FORMAT AND EXPORT TABLES 2 AND 3

table2_gt <- anxiety_table %>%
  gt(
    groupname_col = "Outcome"
  ) %>%

  tab_header(
    title = paste(
      "Table 2. Anxiety and Cost-Related",
      "Medication Nonadherence"
    )
  ) %>%

  tab_source_note(
```

```r
    source_note = paste(
      "Note. Anxiety indicates a positive GAD-2 screen.",
      "Models adjusted for age, sex, race, ethnicity, education,",
      "income-to-poverty ratio, BMI, and perceived health.",
      "The first three outcomes were restricted to prescription",
      "medication users. The final outcome included all adults",
      "with chronic pain. Analyses accounted for the complex",
      "NHIS sampling design."
    )
  )


table3_gt <- depression_table %>%
  gt(
    groupname_col = "Outcome"
  ) %>%

  tab_header(
    title = paste(
      "Table 3. Depression and Cost-Related",
      "Medication Nonadherence"
    )
  ) %>%

  tab_source_note(
    source_note = paste(
      "Note. Depression indicates a positive PHQ-2 screen.",
      "Models adjusted for age, sex, race, ethnicity, education,",
      "income-to-poverty ratio, BMI, and perceived health.",
      "The first three outcomes were restricted to prescription",
      "medication users. The final outcome included all adults",
      "with chronic pain. Analyses accounted for the complex",
      "NHIS sampling design."
    )
  )


table2_gt
table3_gt


gtsave(
  table2_gt,
  "Table2_Anxiety_Regression_Models.docx"
)

gtsave(
  table3_gt,
  "Table3_Depression_Regression_Models.docx"
)


### WEIGHTED OUTCOME PERCENTAGES BY DEPRESSION
```

```r
# Row percentages:
# percent endorsing each outcome within depression groups

dep_skip_pct <- prop.table(
  svytable(
    ~phqscreen + skipmed,
    design = medication_user_design
  ),
  margin = 1
) * 100

dep_less_pct <- prop.table(
  svytable(
    ~phqscreen + lessmed,
    design = medication_user_design
  ),
  margin = 1
) * 100

dep_delay_pct <- prop.table(
  svytable(
    ~phqscreen + delaymed,
    design = medication_user_design
  ),
  margin = 1
) * 100

dep_nomed_pct <- prop.table(
  svytable(
    ~phqscreen + nomed,
    design = chronic_pain_design
  ),
  margin = 1
) * 100


# Display all two-way percentage tables
round(dep_skip_pct, 1)
round(dep_less_pct, 1)
round(dep_delay_pct, 1)
round(dep_nomed_pct, 1)


# Combined depression percentages
depression_percentages <- data.frame(
  Outcome = c(
    "Skipped medication",
    "Took less medication",
    "Delayed filling prescription",
    "Did not get needed medication"
  ),

  `No Depression` = c(
```

```r
    dep_skip_pct["Negative", "1"],
    dep_less_pct["Negative", "1"],
    dep_delay_pct["Negative", "1"],
    dep_nomed_pct["Negative", "1"]
  ),

  Depression = c(
    dep_skip_pct["Positive", "1"],
    dep_less_pct["Positive", "1"],
    dep_delay_pct["Positive", "1"],
    dep_nomed_pct["Positive", "1"]
  ),

  check.names = FALSE
)

depression_percentages$`No Depression` <- round(
  depression_percentages$`No Depression`,
  1
)

depression_percentages$Depression <- round(
  depression_percentages$Depression,
  1
)

depression_percentages

### WEIGHTED OUTCOME PERCENTAGES BY ANXIETY
# Row percentages:
# percent endorsing each outcome within anxiety groups

anx_skip_pct <- prop.table(
  svytable(
    ~gadscreen + skipmed,
    design = medication_user_design
  ),
  margin = 1
) * 100

anx_less_pct <- prop.table(
  svytable(
    ~gadscreen + lessmed,
    design = medication_user_design
  ),
  margin = 1
) * 100

anx_delay_pct <- prop.table(
  svytable(
    ~gadscreen + delaymed,
    design = medication_user_design
  ),
```

```r
  margin = 1
) * 100

anx_nomed_pct <- prop.table(
  svytable(
    ~gadscreen + nomed,
    design = chronic_pain_design
  ),
  margin = 1
) * 100


# Display all two-way percentage tables
round(anx_skip_pct, 1)
round(anx_less_pct, 1)
round(anx_delay_pct, 1)
round(anx_nomed_pct, 1)


# Combined anxiety percentages
anxiety_percentages <- data.frame(
  Outcome = c(
    "Skipped medication",
    "Took less medication",
    "Delayed filling prescription",
    "Did not get needed medication"
  ),

  `No Anxiety` = c(
    anx_skip_pct["Negative", "1"],
    anx_less_pct["Negative", "1"],
    anx_delay_pct["Negative", "1"],
    anx_nomed_pct["Negative", "1"]
  ),

  Anxiety = c(
    anx_skip_pct["Positive", "1"],
    anx_less_pct["Positive", "1"],
    anx_delay_pct["Positive", "1"],
    anx_nomed_pct["Positive", "1"]
  ),

  check.names = FALSE
)

anxiety_percentages$`No Anxiety` <- round(
  anxiety_percentages$`No Anxiety`,
  1
)

anxiety_percentages$Anxiety <- round(
  anxiety_percentages$Anxiety,
  1
```

```
)

anxiety_percentages
```
